# Benzimidazole -Resistance in *Haemonchus contortus*: New PCR-RFLP Method for the Detection of Point Mutation at Codon 167 of Isotype 1 β-Tubulin Gene

**Published:** 2012

**Authors:** HR Shokrani, P Shayan, A Eslami, R Nabavi

**Affiliations:** 1 Department of Parasitology, Faculty of Veterinary Medicine, University of Tehran, Iran; 2 Department of Parasitology, Faculty of Veterinary Medicine, University of Zabol, Iran

**Keywords:** *Haemonchus contortus*, Benzimidazole resistance, β-Tubulin, Point mutation, PCR-RFLP

## Abstract

**Background:**

Due to the lack of a suitable and economic test for the analysis of the polymorphism at codon 167, we developed a new PCR-RFLP technique, based on a modified forward primer (UT-HC167 MF-primer), to identify simultaneously the SNPs at codons 167 and 200 of isotype 1 β-tubulin gene of *Haemonchus contortus*.

**Methods:**

There already are several safe and easy methods for identification of point mutations at codons 198 and 200. Due to the lack of a reliable and easy method for the detection of the single nucleotide polymorphism (SNP) at codon 167, we developed an innovative PCR-RFLP technique based on a modified forward primer (UT-HC167 MF-primer), in which the nucleotide T at the position 443 was substituted through a nucleotide A creating a restriction site for restriction endonuclease SnaB I in the nucleotide sequences including codon 167. A total of 138 adult male *H. contortus* were collected from three different geo-climatic areas of Iran. The isolated genomic DNA of each single worm was amplified by PCR using primers flanking codon 167. The PCR product (527 bp) was then amplified by semi-nested PCR using the UT-HC167 MF-primer and the reverse primer achieving a PCR product of 451 bp in length. This PCR product was subsequently digested with the restriction endonucleases SnaB I and TaaI for analysis of the mutations at codons 167 and 200, respectively.

**Results:**

All worms had two alleles encoding for phenylalanine (BZ^ss^ homozygote) for both codons.

**Conclusion:**

Using the UT-HC167 MF-primer and a suitable reverse primer designed upstream from codon 200, it is possible to amplify a PCR product which can be used for analysis of the SNPs at all three mentioned codons using RFLP.

## Introduction

*Haemonchus contortus* is the most pathogenic abomasal parasite of small ruminants. In Iran, next to *Marshallagia marshalli* and *Teladorsagia circumcincta, H. contortus* is the most prevalent species of gastrointestinal nematodes of sheep and goats. The infection is currently controlled by three major classes of anthelmintics: benzimidazoles (BZs), imidazothiazoles and macrocyclic lactones. BZs bind selectively to the cellular β-tubulin of helminthes leading to the inhibition of microtubule formation (1). Because of high efficiency and absence of toxic residues, BZs especially albendazole are the most commonly used anthelmintic drug in Iran.

It was shown that BZ-resistance is associated with point mutations in β-tubulin gene that prevent drug binding. Genetic analysis revealed that the SNPs at codons 167, 198 or 200 of isotype 1 β-tubulin gene are responsible for the resistance development against BZs in helminthes. In trichostrongylid of small ruminants the well-known molecular mechanism that confers BZ-resistance involves the SNP at position 200, which leads to substitution of phenylalanine into tyrosine (2–5). Another mutation at codon 167 of isotype 1 β-tubulin gene which leads to the same substitution was detected in BZ-resistant populations of some nematodes such as *H. contortus* and *T. circumcincta* in small ruminants and cyathostomins in horses (6–9). More recently, a third mutation was detected at codon 198 of isotype 1 β-tubulin gene of *H. contortus* leading the substitution of glutamate into alanine, causing resistance against BZs (10).

BZ-resistance in nematodes has been reported using different biological and molecular methods (11, 12). Several PCR based tests have been developed for the detection of BZ-resistance (13). These tests only can be used to detect one point mutation (mostly the SNP at codon 200). If the SNPs other than those at codon 200 are present, current tests will not detect the resistance. Furthermore, some molecular methods such as the classical sequencing and the pyrosequencing assays are expensive to routine use.

Due to the lack of a suitable and economic test for the analysis of the polymorphism at codon 167, we developed a new PCR-RFLP technique, based on a modified forward primer (UT-HC167 MF-primer), to identify simultaneously the SNPs at codons 167 and 200 of isotype 1 β-tubulin gene of *H. contortus*.

## Materials and Methods

### Parasite collection & DNA extraction

Nematodes were collected from local abattoirs directly from sheep and goats abomasa from three different geo-climatic areas of Iran, Babol (in north, humid area), Chadegan (in center, mountain area) and Shooshtar (in south, sultry area). The adult worms were identified morphologically according to Skrjabin (14). A total of 138 adult male *H. contortus*, 46 from each area, were collected in separate tubes containing 70% ethanol.

DNA extraction was performed using a DNA isolation kit (MBST, Iran) according to the manufacturer's instructions. Briefly, each single worm was lysed in 180 µl lysis buffer and 20 µl proteinase K for 30 min at 55°C. After adding 360 µl binding buffer and incubation for 10 min at 70°C, 270 µl ethanol (100%) was added to the solution. After vortexing, the complete volume was transferred to the MBST column. The MBST column was first centrifuged and then washed twice with 500 µl washing buffer. Finally, DNA was eluted from the carrier with 100 µl elution buffer.

### Primer design

**Fig. 1:**
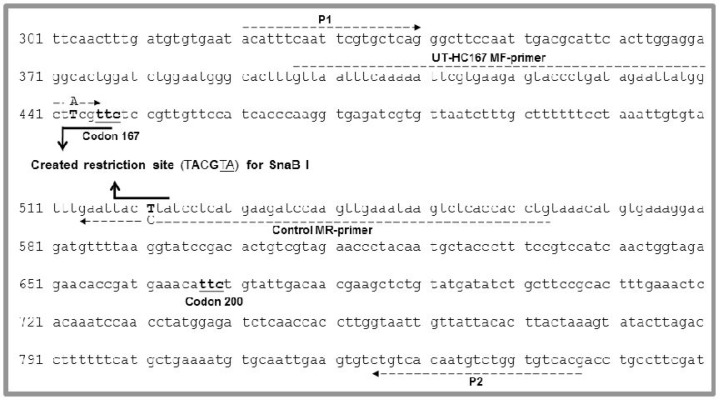
UT-HC167 MF-primer was designed from nucleotide in positions 397 to 446, in which at the position 443 the nucleotide T was substituted through a nucleotide A to create a restriction site for SnaB I. Furthermore, to create a positive control template for SnaB I, Control MR-primer was designed from nucleotide in positions 514 to 563, in which at the position 521 the nucleotide A was substituted through a nucleotide C. The arrows show the forward and reverse primers annealed to the complementary sequence of isotype 1 β-tubulin gene of *H. contortus* (accession: X80046; version X80046.1 GI: 897752)

Primers were designed from *H. contortus* isotype 1 β-tubulin gene (accession: X80046; version X80046.1 GI: 897752). The forward primer (P1) was from nucleotide in positions 321 to 340 (20-mer) and the reverse primer (P2) was from nucleotide in positions 825 to 847 (23-mer). For semi-nested PCR, we designed a modified forward primer (UT-HC167 MF-primer) from nucleotide in positions 397 to 446 (50-mer), in which at the position 443 the nucleotide T was substituted through a nucleotide A to create a restriction site (T**A**CGTA) for the restriction endonuclease SnaB I. This restriction site included the first two nucleotide sequences coding for tyrosine (TAC) at codon 167 (Fig. 1; Table 1).

**Table 1:** Details of the PCR primers that were designed from isotype 1 β-tubulin gene of *H. contortus* (accession: X80046; version X80046.1 GI: 897752). In UT-HC167 MF-primer the nucleotide T was replaced with the underlined nucleotide A. Furthermore, in Control MR-primer the nucleotide A was replaced with the underlined nucleotide C.

Name of reaction	Name of primer	Sequence (5’–3’)	PCR product
PCR	P1 (Forward)	acatttcaattcgtgctcag	Ca. 527 bp
	P2 (Reverse)	cgtgacaccagacattgtgacag	
Semi-nested PCR	UT-HC167 MF-primer	gttaatttcaaaaattcgtgaagagtaccctgatagaattatggct**A**cgt	Ca. 451 bp
	P2	cgtgacaccagacattgtgacag	
Enzyme-control PCR	P1	acatttcaattcgtgctcag	Ca. 243 bp
	Control MR-primer	caggtggtgagacttatttcaacttggatcttcatgaggata**C**gtaattc	

To create a positive control for SnaB I, we also designed a modified reverse primer (Control MR-primer) from nucleotide in positions 514 to 563 (50-mer), in which at the position 521 the nucleotide A was substituted through a nucleotide C (Fig. 1; Table 1).

### Polymerase chain reaction (PCR) & semi-nested PCR

PCR was carried out on 5 µl of DNA solution in a total volume of 50 µl, including one time PCR buffer, 1.5 U Taq DNA polymerase, 1.5 mM MgCl2, 1 µl of each primer (P1 and P2, 20 mM each, Cina Gene, Iran) and 100 µM of each dATP, dTTP, dCTP and dGTP (Fermenta), using a Bio-Rad thermal cycler. The following program was performed: initial denaturation at 95 °C for 5 min; 38 cycles of 45 s at 94°C, 45 s at 56°C and 60 s at 72°C; final extension at 72°C for 10 min. As a negative template control distilled water was used. The PCR product analysis was done by gel electrophoresis. A 527 bp fragment was amplified. The PCR products were purified with a PCR purification kit (MBST, Iran) according to the manufacturer's instructions. Briefly, 200 µl binding buffer was added to 100 µl PCR product solution. After adding 150 µl ethanol (96%) to the sample, the mixture was applied into the column. The column was washed twice with washing buffer, and PCR product was eluted from the column using 100 µl elution buffer.

Semi-nested PCR was performed to control the specificity of the first PCR products, also to create a recognition site for SnaB I using the new modified forward primer (UT-HC167 MF-primer). It was carried out on 1-5 µl of each PCR product in a total volume of 50 µl. The primers used for semi-nested PCR were UT-HC167 MF-primer and P2 primer. The following cycling conditions were performed: initial denaturation at 95 °C for 5 min; 38 cycles including 2 cycles of 45 s at 94°C, 90 s at 66°C and 60 s at 72°C, 2 cycles of 45 s at 94°C, 60 s at 66°C and 60 s at 72°C and 34 cycles of 45 s at 94°C, 45 s at 66°C and 60 s at 72°C; final extension at 72°C for 10 min. After amplification, the PCR products were analyzed on 1.5% agarose gel. A 451 bp fragment was amplified. Subsequently, these PCR products were purified for enzyme digestion using MBST PCR purification kit.

An additional PCR reaction was performed using P1 primer and Control MR-primer to create a PCR product having a restriction site for SnaB I. PCR was carried out on 5 µl of DNA solution in a total volume of 50 µl. The following cycling conditions were performed: initial denaturation at 95 °C for 5 min; 38 cycles including 2 cycles of 45 s at 94°C, 90 s at 56°C and 60 s at 72°C, 4 cycles of 45 s at 94°C, 60 s at 56°C and 60 s at 72°C and 32 cycles of 45 s at 94°C, 45 s at 56°C and 60 s at 72°C; final extension at 72°C for 10 min. The resulted PCR product of 243 bp was used as a positive control template for SnaB I activity.

### Detection of point mutation at codon 167

Fifteen µl of each purified PCR product (451 bp, approximately 200 ng) was digested with 1 U SnaB I (Roche, 10 U/µl) in 2.5 µl 10× SnaB I buffer and 7.4 µl distilled water for 1 h at 37°C. The RFLP reactions were then analyzed on 2% agarose gel. As a negative control, 15 µl of PCR product was treated with 2.5 µl 10× SnaB I buffer and 7.4 µl distilled water without adding enzyme. To control the enzyme activity, the PCR product of 243 bp was used as a positive control template.

### Detection of point mutation at codon 200

The PCR products (451 bp) were also digested with TaaI (Fermenta, 10 U/µl) to analyze the SNP at codon 200 (15). The fragments were distinguished from each other on 2% agarose gel. Beside the restriction site at codon 200, there is a second restriction site for TaaI at base 603 that creates an additional fragment (207 bp) providing an internal positive control for TaaI. Nevertheless, the additional fragment does not interfere with explanation of results.

## Results

**Fig. 2:**
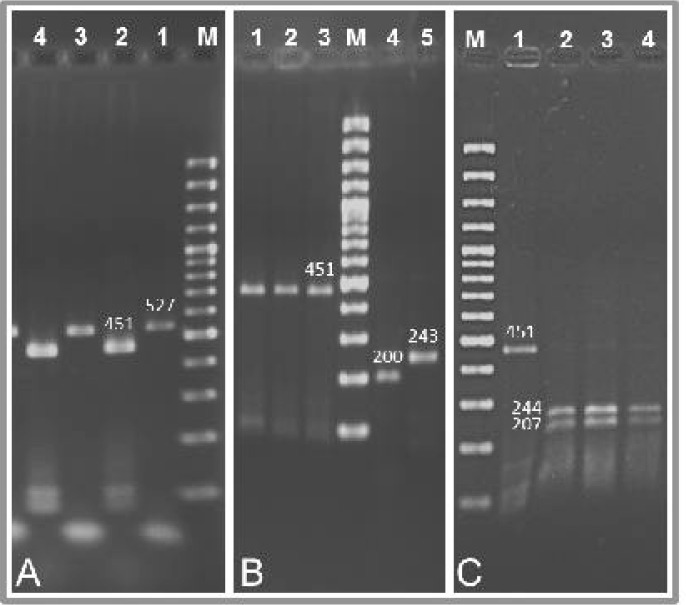
**A** Genomic DNA of each single *H. contortus* was amplified with P1 and P2 primers to obtain a PCR product of 527 bp (Lane 1 and 3). Subsequently, each PCR product was amplified with UT-HC167 MF-primer and P2 primer to obtain a PCR product of 451 bp containing codons 167 and 200 (Lane 2 and 4). **B** The second PCR products were digested with SnaB I. The products could not be cut with this enzyme (BZ^SS^; Lane 1, 2 and 3). To control the activity of enzyme, the positive control template (243 bp; Lane 5) was cut into two fragments of 200 and 43 bp (Lane 4). **C** The second PCR products (451 bp; Lane 1) were also digested with TaaI. The products were cut into two fragments of 207 and 244 bp (BZ^SS^; Lane 2, 3 and 4). M is 100 bp marker

Hundred thirty eight adult male *H. contortus* (3 worms/animal; 46 worms/area) were genotyped for the detection of point mutations at codons 167 and 200 of isotype 1 β-tubulin gene. Only adult male worms were used in order to avoid the risk of unreliable DNA amplification from the eggs of female worms (16). The isolated genomic DNA of each single worm was separately used to amplify the isotype 1 β-tubulin gene. The isotype 1 β-tubulin specific PCR product showed an expected fragment of 527 bp in length (Fig. 2A).

Since the SNP at codon 167 was not detectable by PCR-RFLP, a modified forward primer (UT-HC167 MF-primer) was designed creating a restriction site for SnaB I. Each PCR product (527 bp) was amplified with the modified forward primer and P2 primer resulted an expected fragment of 451 bp containing codons 167 and 200 (Fig. 2A). Reproducibility of the results showed that the substitution of one nucleotide had no negative effect on annealing of the modified forward primer. The PCR product (451 bp) was subsequently digested with SnaB I to detect the SNP at codon 167. The mutated allele (TAC) can be cut into two fragments (403 and 48 bp), whereas the normal allele (TTC) cannot be cut with SnaB I.

The DNA solutions extracted from 138 single worms were analyzed for the SNP at codon 167. The RFLP analysis showed that all 138 worms had two alleles encoding for phenylalanine (BZ^SS^ homozygote). No resistant allele (BZ^RS^ heterozygote or BZ^RR^ homozygote) was demonstrable at this codon (Fig. 2B).

To control the activity of enzyme, an additional PCR reaction was performed using a modified reverse primer (Control MR-primer) and P1 primer creating a 243 bp fragment having a restriction site for SnaB I. The PCR product was subsequently digested with SnaB I cutting into two fragments of 200 and 43 bp (Fig. 2B). These results confirmed the activity of SnaB I and also showed the digestibility of the PCR product amplified by a modified primer.

The semi-nested PCR product was also treated for the SNP at codon 200 using the restriction endonuclease TaaI. A second restriction site (ACTGT) existed between nucleotide in positions 601 to 605, made it possible to analyze simultaneously the SNP at codon 200 and the enzyme activity. In the case of normal allele (TTC), TaaI cut the PCR product (451 bp) into two fragments (207 and 244 bp), whereas in the case of mutated allele (TAC), TaaI cut the PCR product into three fragments (207, 177 and 67 bp). The DNA solutions extracted from 138 single worms were digested with TaaI cleaved into two fragments of 207 and 244 bp (Fig. 2C). The results showed that all worms had two alleles encoding for phenylalanine (BZ^SS^ homozygote) at codon 200 and no resistant allele (BZ^RS^ heterozygote or BZ^RR^ homozygote) was demonstrable in this codon.

In an additional experiment, approximately a third of DNA samples (45 samples) were amplified directly using UT-HC167 MF-primer and P2 primer. The results showed that it is possible to amplify the corresponding DNA fragment without need to the first PCR reaction. Although nested PCR involves two amplification steps and potentially improves both sensitivity and specificity, one PCR reaction can also be used for epidemiological studies dealing with SNPs analysis.

## Discussion

Resistance to benzimidazole in trichostrongylid nematodes is associated with several independent point mutations of the β-tubulin genes. The SNPs at codon 200, codon 167 or codon 198 of isotype 1 β-tubulin gene are known to be associated with BZ-resistance in *H. contortus*. It is believed that the SNP at codon 200 is the most important mutation conferring BZ-resistance in gastrointestinal nematodes of small ruminants. Binding studies with recombinant *H. contortus* β-tubulins indicated that the point mutations at codon 167 of β-tubulin genes reduce affinity for benzimidazoles (6). The significance of the SNP at codon 167 for different parasites has been only partially investigated (17). Silvestre and Cabaret (7) determined the SNP at codon 167 in *T. circumcincta* and *H. contortus* by sequencing of the PCR products containing codon 167. They described that the SNP at codon 167, may participate in BZ-resistance in trichostrongylid nematodes, in the absence of the mutation at codon 200. Several molecular methods such as allele-specific PCR (AS-PCR) and PCR-restriction fragment length polymorphism (PCR-RFLP) have been explored for *H. contortus* which can detect point mutations at codons 200 or 198 of isotype 1 β-tubulin gene (2, 18, 19, 15, 10, 20).

Although AS-PCR is an excellent and sensitive method, in our knowledge, there is not AS-PCR method described for the analysis of the SNP at codon 167. This might be associated with specific difficulties encountered during the establishment of the mentioned method. Furthermore, due to the lack of any restriction site with respect to the codon 167, the analysis of the SNP at this codon is more complex than other SNPs at codons 198 and 200.

In the present study we described an innovative PCR-RFLP method which can be used for rapid and easy identification of the SNP at codon 167 of isotype 1 β-tubulin gene of *H. contortus*. For this aim a modified forward primer (UT-HC167 MF-primer) was designed to create a recognition site for the restriction endonuclease SnaB I. The last two nucleotide sequences of the created restriction site belong to the first two nucleotides coding for tyrosine (TAC) at codon 167. Additionally, the PCR product is also informative for the SNP analysis at codon 200, which is easily demonstrable using the restriction endonuclease TaaI. The PCR product is long enough (451 bp) to obtain a clear effect from the both restriction endonucleases SnaB I and TaaI.

Recently, a pyrosequencing based method was described for the quantitative analysis of the point mutations within the β-tubulin gene. This method is based on the sequencing of the target DNA. The investigations showed that the SNPs at codons 167, 198 and 200 of isotype 1 β-tubulin gene of *H. contortus* can also be detected via this method (21, 22).

BZ-resistance in *H. contortus* has become a major problem in sheep and goats industry of many countries located in tropics and subtropics areas. We analyzed the SNP at codons 167 and 200 in 138 single *H. contortus*. No mutation could be detected in analyzed worms collected from three different geo-climatic areas of the country. All worms were homozygous susceptible (BZ^SS^) at both codons.

There are a few investigations about the status of BZ-resistance in nematode populations of Iran. These results were in agreement with results obtained by Gholamian et al. (23) who could not detect BZ-resistance in *H. contortus* using faecal egg count reduction test (FECRT). Nevertheless, in Iran, resistance against BZs was already demonstrated in *T. circumcincta* using PCR-RFLP (24). The high frequency of susceptible allele (BZ^SS^) in *H. contortus* populations might be due to the severe drought over the last decade in the country which is detrimental for free-living stages of the nematodes especially *H. contortus*. The drought has caused low nematode infections and thereby lesser use of anthelmintics by livestock owners. It is generally recognized that the frequency of treatment is a major driver of the development of anthelmintic resistance in nematode populations. Taylor et al. (25) described truly that in the Middle East countries, such as Iran, the survival of *H. contortus* is associated with the ability of hypobiosis by the parasite larvae. Resumption of development occurs just before the onset of seasonal rains. Due to the climate changes in all regions of the country especially low rainfall over the last decade, the *H. contortus* infestation in small ruminants of Iran is lower than other gastrointestinal nematodes such as *T. circumcincta* (26), thus the number of generations, the rate of expose to BZs and in consequence selection pressures to resistant populations of *H. contortus* is lower than other gastrointestinal nematode populations especially *T. circumcincta*.

Early detection of BZ-resistance is a crucial point for farms management and application for prevention strategies. Samson-Himmelstjerna (13) described that it is important to maintain the efficacy of currently available anthelmintics wherever resistance has not emerged. It seems that if the BZ-resistant nematodes reach to medium level in natural population, no reversion is possible, even if another anthelmintic molecule without any cross-resistance is used (27). Therefore, although resistance against BZs in gastrointestinal nematodes of small ruminants of Iran occurs at a low prevalence, the periodic investigations for early detection of mutated alleles in nematode populations using rapid and sensitive molecular methods such as PCR-RFLP will be of great importance for controlling of BZ-resistance in Iran.

## Conclusion

The innovative PCR-RFLP technique described in this paper is an easy, rapid and sensitive method to amplify a PCR product containing codons 167, 198 and 200 which can be used simultaneously for analysis of the SNPs at all three mentioned codons using three suitable restriction endonucleases.
